# Rare Coexistence of Giant Cell Tumor and Tuberculosis of the Metatarsal

**DOI:** 10.7759/cureus.12090

**Published:** 2020-12-15

**Authors:** Febin Kunnath, Kaushik Bhowmick, Boopalan P.R.J.V.C

**Affiliations:** 1 Orthopaedics, Christian Medical College and Hospital, Vellore, IND

**Keywords:** giant cell tumor (gct), metatarsal bone, tuberculosis (tb)

## Abstract

The coexistence of giant cell tumor (GCT) and metatarsal bone tuberculosis (TB) of the foot has not been reported in the literature so far. We report a case of a 25-year-old male who presented with severe pain and swelling of his left foot for two months, which was aggravated on walking. A plain radiograph of the left foot showed an expansile eccentric lytic lesion of the base of the second metatarsal. He underwent extended curettage and antibiotic cement spacer insertion. Biopsy of the lesion revealed the presence of GCT, while tissue cultures were positive for Mycobacterium tuberculosis. He was treated with standard anti-tubercular treatment (ATT), four drug regimens for twelve months. He then underwent reconstruction of the second metatarsal with cement spacer exit and iliac crest bone grafting, following which the cultures were negative for TB. The diagnosis of this unexpected and unique combination of pathologies (GCT and TB) depends on a high index of clinical suspicion, relevant investigations, and accurate histological diagnosis.

## Introduction

Giant cell tumor (GCT) of bone is a benign but locally aggressive tumor with a recurrence rate of up to 50% [1, 2]. The most frequent locations, in decreasing order, are the distal femur, the proximal tibia, the distal radius, and the proximal humerus [3]. It usually occurs in young adults of 20 to 40 years of age in the epiphysio-metaphyseal region with a female preponderance. Involvement of the bones of the hand and foot is rare, with incidences ranging 2-4% in hand and 1.2-1.8% in the foot. GCT of the hand and foot has a high incidence of multicentricity, appears at a younger age, and has a shorter duration of symptoms. GCT of the extremities is usually radiologically aggressive. The most common symptoms are pain and swelling of the involved region [4-6].

Osteoarticular TB makes up to less than 3% of the cases of active pulmonary TB, of which about 10% involve foot and ankle [2]. Foot TB may become significantly debilitating if left untreated or if the diagnosis is delayed. Mittal et al. classified TB of the foot into five radiological types: cystic, rheumatoid, sub-periosteal, kissing, and spina ventosa [7]. Most of the lesions show clinical and radiological healing with anti-tubercular therapy [7, 8].

In this case report, we present the first observation of the coexistence of GCT and tuberculosis of the metatarsal bone.

## Case presentation

**Figure 1:**
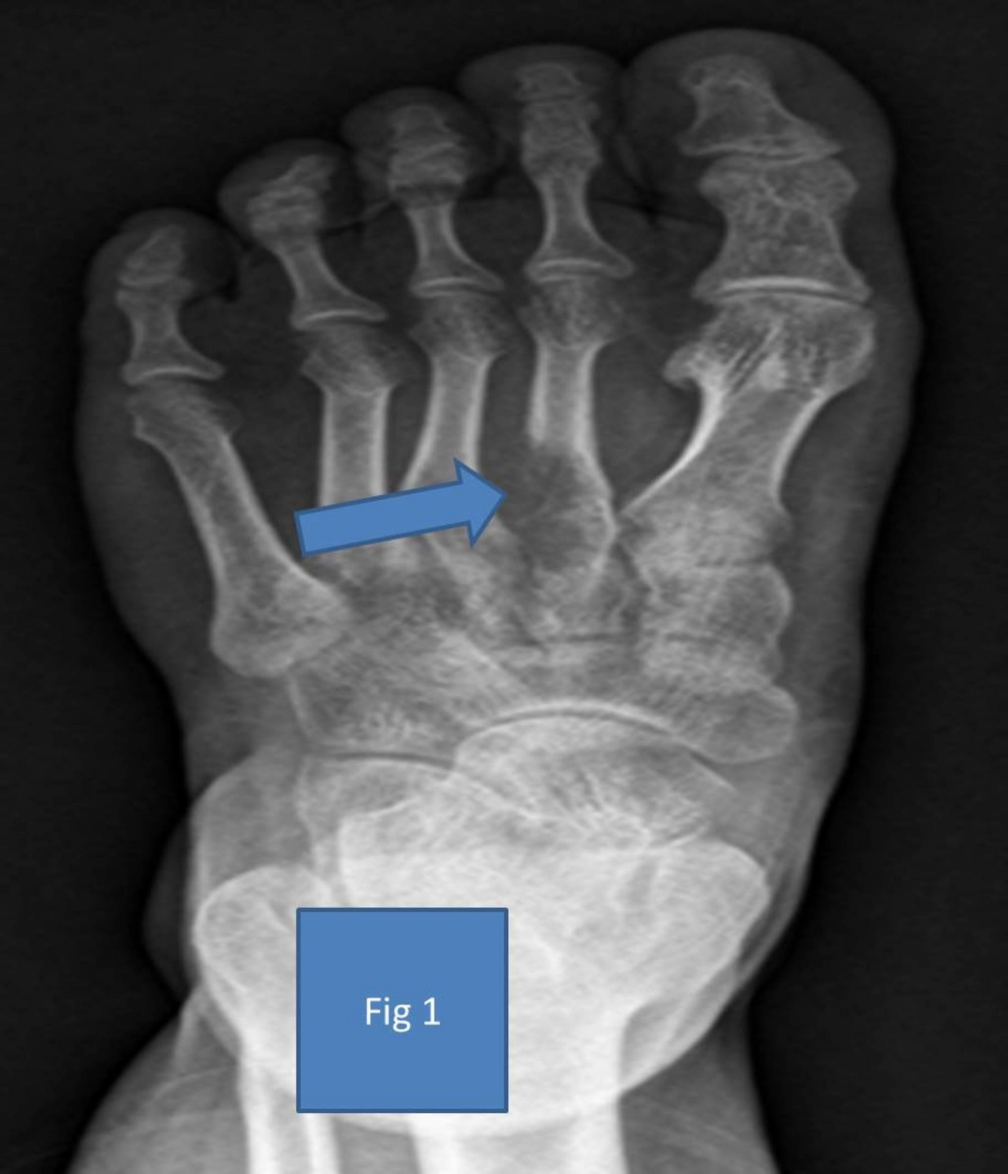
Pre-operative radiograph showing a lytic lesion in the epiphysio-metaphyseal region of the second metatarsal

A 25-year-old male presented with a two-month history of pain and swelling of the left foot to Christian Medical College and Hospital, Vellore, India. The swelling was insidious in onset, progressive, associated with pain and difficulty in walking. There was no past or family history of tuberculosis. On examination, he had tenderness and diffuse swelling over the left second ray. There were no associated skin changes. A plain radiograph of the left foot showed an expansile lytic lesion of the metaphyseo-diaphyseal region of the base of the second metatarsal bone (Figure 1). Bone scintigraphy showed an increased tracer activity in the second metatarsal base without the involvement of any other region of the body. Blood parameters, which included calcium/phosphorus/alkaline phosphatase/parathyroid hormone, were within normal limits. The inflammatory markers were not done initially.

Surgical procedure

**Figure 2:**
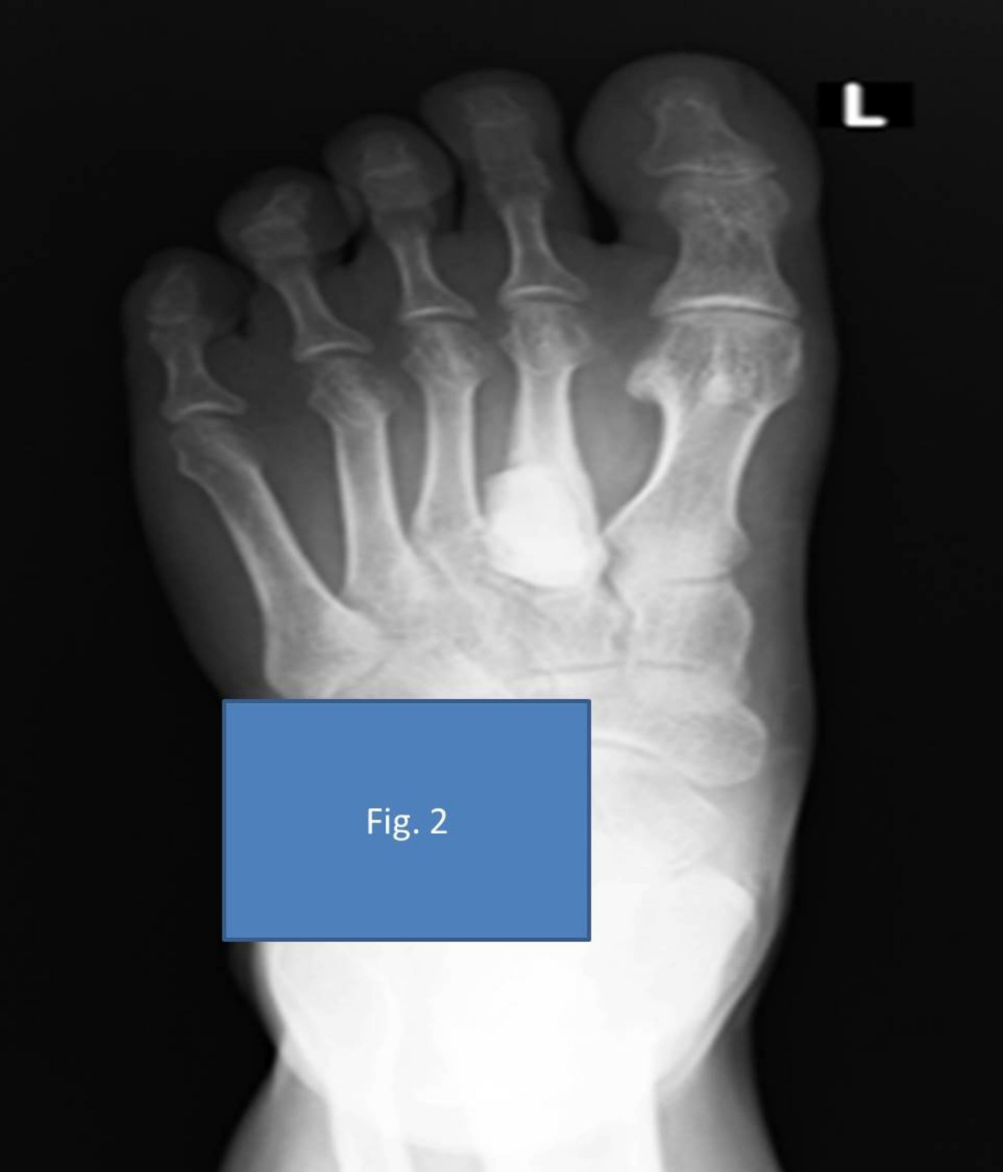
Extended curettage and cement spacer application on the second metatarsal anteroposterior (AP) view

**Figure 3:**
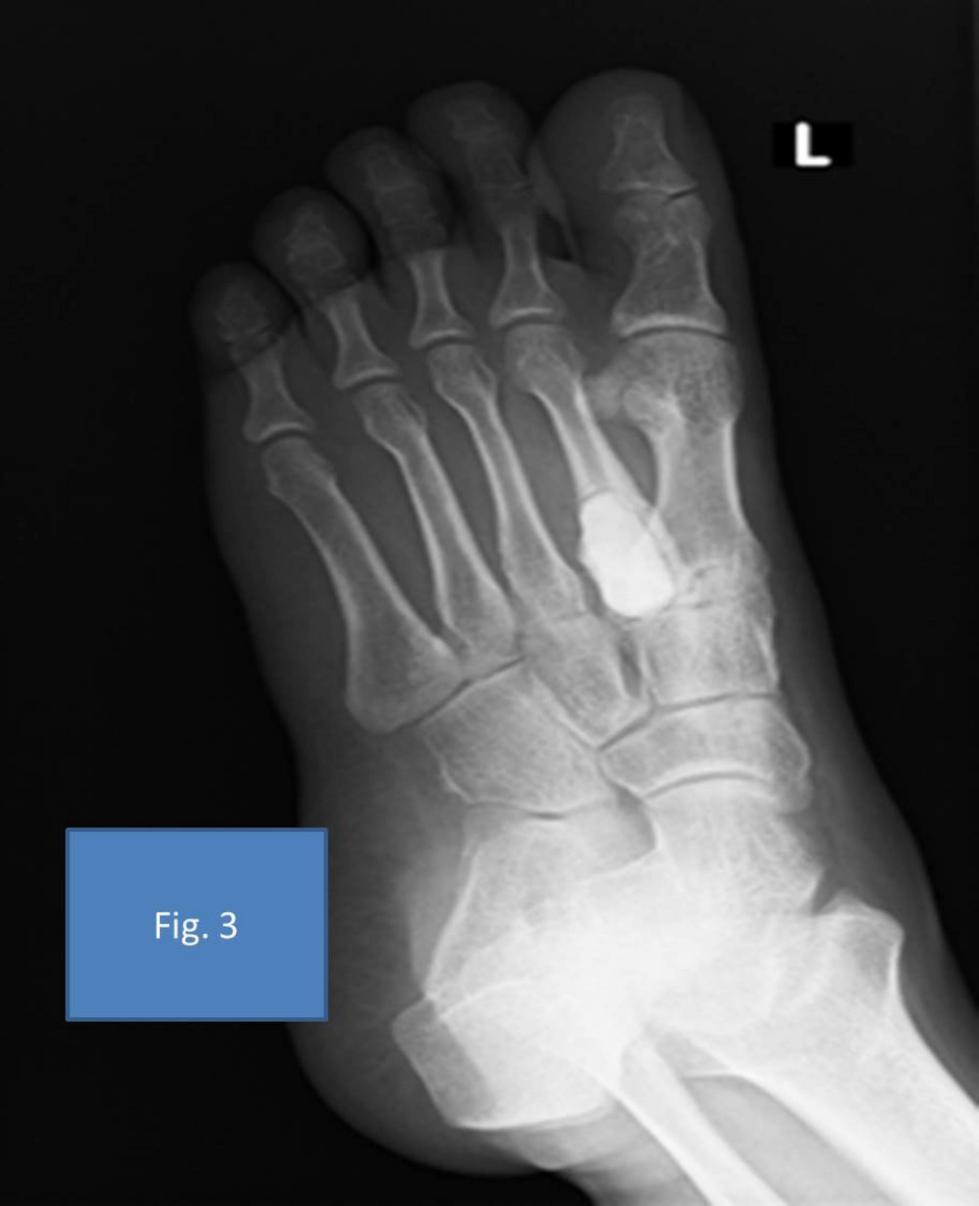
Extended curettage and cement spacer application on the second metatarsal oblique view

Staged treatment was planned for the patient. In the first stage, he underwent excision biopsy of the lesion, extended curettage with speed burr, and chemical cauterization with 80% phenol. The curetted area was filled with antibiotic-loaded bone cement consisting of 1 gram each of vancomycin and meropenem (Figures 2-3). Perioperatively, the lesion was appearing like an atypical GCT. The tissue sample was sent for bacterial and fungal cultures and biopsy. The biopsy was reported as a giant cell-rich lesion, consistent with GCT. The GeneXpert® TB polymerase chain reaction (PCR) test detected Mycobacterium tuberculosis from the tissue sample without rifampicin resistance. He was started on treatment for tuberculosis by a standard four-drug protocol of isoniazid, rifampicin, pyrazinamide, and ethambutol for two months in the intensive phase and isoniazid and rifampicin for 10 months in the continuous phase. Pyridoxine was given along with these drugs for the same period.

The second stage of treatment was started after the completion of the full course of anti-tubercular therapy. The metatarsal base was again exposed through the previous incision, and the cement bead was removed. Swabs were taken from the area and sent for cultures. The defect at the metaphyseo-diaphyseal part was measured, and the cortico-cancellous graft was taken from the iliac crest and press-fitted into the defect. Cultures were negative for Mycobacterium tuberculosis.

**Figure 4:**
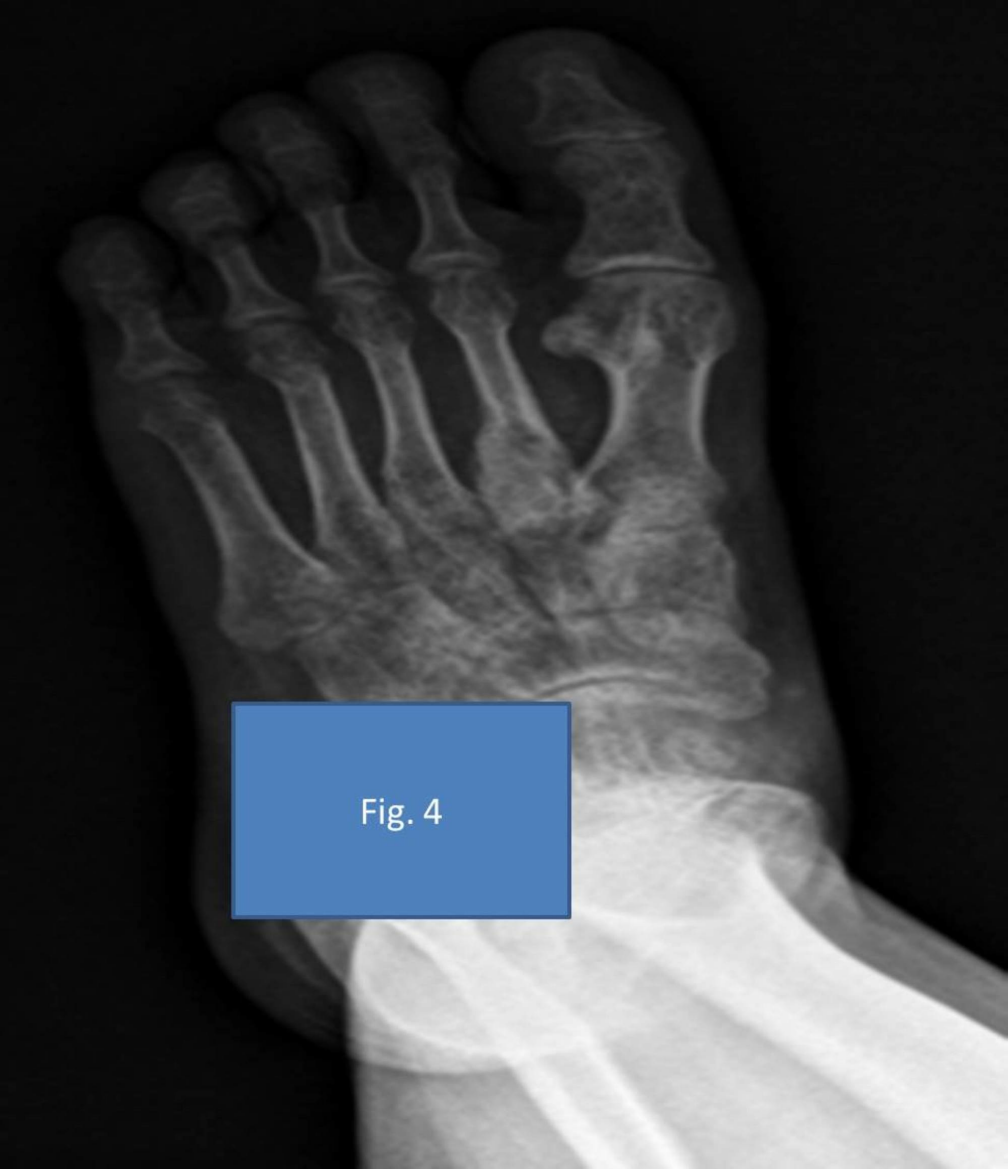
Complete assimilation of the bone graft on the second metatarsal three years post-surgery anteroposterior (AP) view

**Figure 5:**
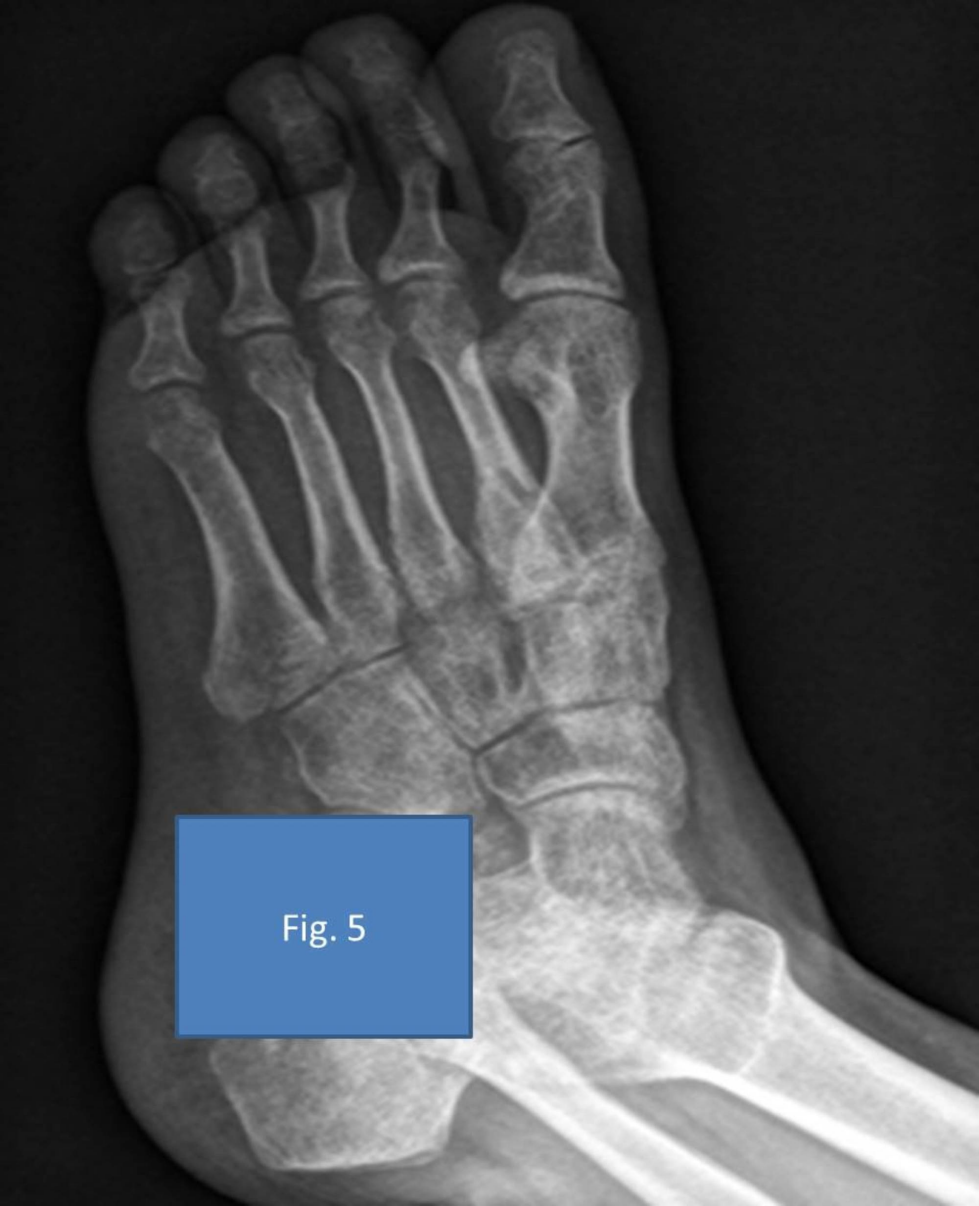
Complete assimilation of the bone graft on the second metatarsal three years post-surgery oblique view

After surgery, the patient was given a below-knee cast and started on weight-bearing as tolerated. The patient was being followed up in the outpatient department (OPD) regularly for three years, and the radiographs showed complete integration of the bone graft without any recurrence to date (Figures 4-5).

## Discussion

The existence of GCT or tuberculosis of the metatarsal bone by itself is very rare. However, both are considered as a differential diagnosis for lytic lesions of the bone in the extremities. Other differential diagnoses are an aneurysmal bone cyst, Brown's tumor, non-ossifying fibroma, chondroblastoma, Langerhans cell histiocytosis, and chronic osteomyelitis [5, 8-10].

The co-existence of GCT and tuberculosis has been reported only in one case report by Charach et al. where the authors have reported this unusual co-existence in the metacarpal bone. In this report, the diagnosis was obtained after an open biopsy, and antitubercular therapy was given for a period of eight months, after which the patient underwent curettage of the metacarpal [11].

**Figure 6:**
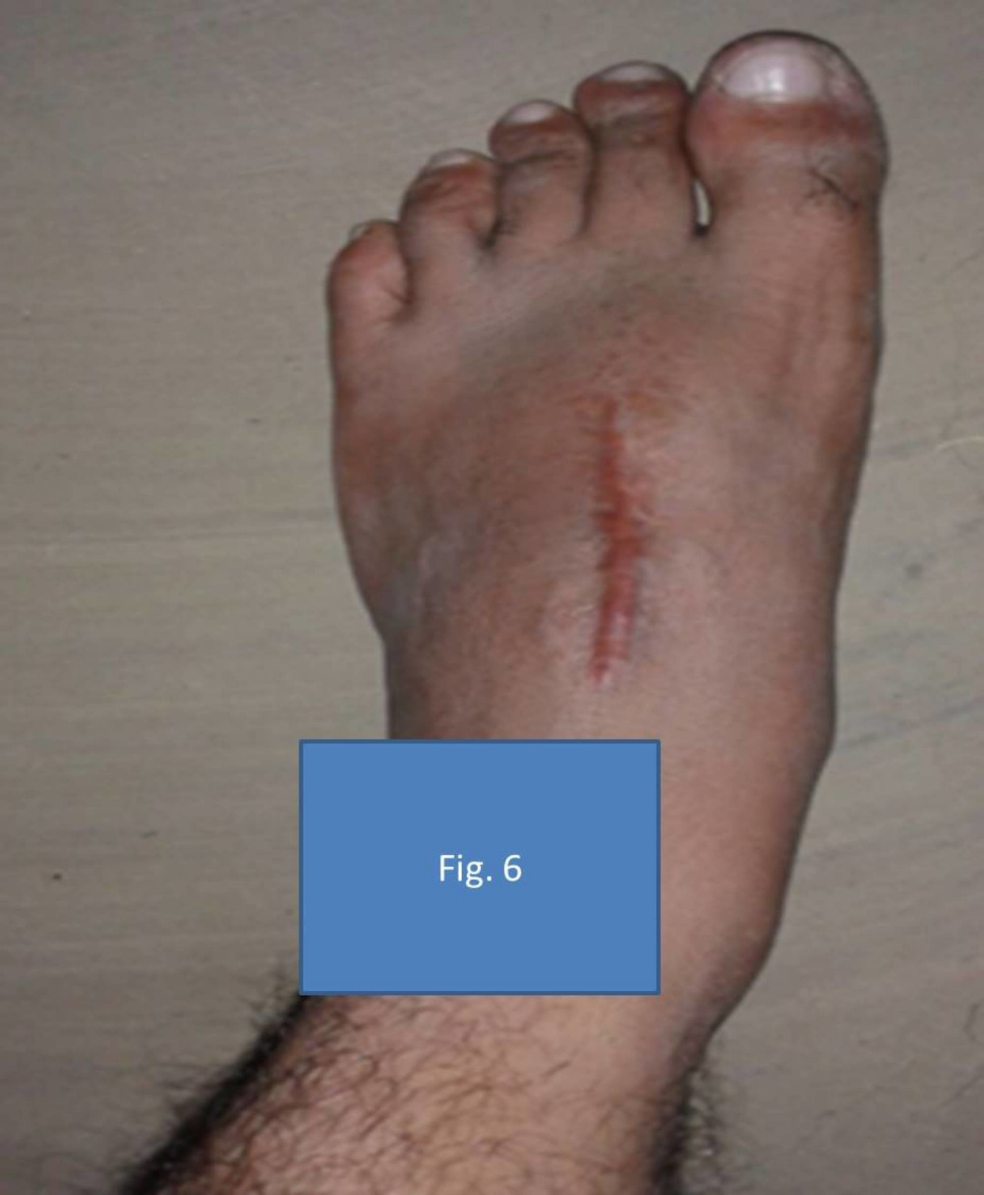
Patient’s foot three years post-surgery

Our patient had a lytic lesion on the metaphyseo-diaphyseal region of the left second metatarsal, which was eccentric and had a breach of cortex on the lateral side. Extended curettage was done, and the gap was filled with antibiotic-loaded bone cement (ALBC). We proceeded with the metatarsal reconstruction only after completion of one year of anti-tubercular therapy and periodic evaluation of inflammatory markers and radiographs to check the resurgence of infection. We were aggressive in doing extended curettage during the first stage of treatment as our first differential diagnosis was GCT. On approaching the lesion, there was minimal granular tissue, because of which we added ALBC instead of adding bone graft after the curettage. Our patient had been followed up for three years now and has no signs of recurrence of either the GCT or infection (Figure 6). His American Orthopaedic Foot and Ankle Society (AOFAS) midfoot score is 88 out of 100 at three years. This score takes into account pain, function, and alignment of the midfoot.

We add to the literature the case of co-existence of GCT and tuberculosis in lytic lesions and hope that our management supplements the treatment principles in these types of patients.

## Conclusions

The rare combination of GCT and infection should be kept in mind while evaluating lytic lesions in the extremities. Tubercular, bacterial, and fungal cultures should always be sent along with the biopsy specimen for a complete evaluation.
